# Oregano essential oil-pectin edible films as anti-*quorum sensing* and food antimicrobial agents

**DOI:** 10.3389/fmicb.2014.00699

**Published:** 2014-12-17

**Authors:** Maria V. Alvarez, Luis A. Ortega-Ramirez, M. Melissa Gutierrez-Pacheco, A. Thalia Bernal-Mercado, Isela Rodriguez-Garcia, Gustavo A. Gonzalez-Aguilar, Alejandra Ponce, Maria del R. Moreira, Sara I. Roura, J. Fernando Ayala-Zavala

**Affiliations:** ^1^Consejo Nacional de Investigaciones Científicas y Técnicas - Grupo de Investigación en Ingeniería en Alimentos, Facultad de Ingeniería, Universidad Nacional de Mar del PlataMar del Plata, Argentina; ^2^Laboratorio de Tecnologías Emergentes, Coordinación de Tecnología de Alimentos de Origen Vegetal, Centro de Investigación en Alimentación y DesarrolloHermosillo, México

**Keywords:** *Chromobacterium violaceum*, food safety, natural compounds, edible coatings, cell communication

## Abstract

Edible films can be used as carriers for antimicrobial compounds to assure food safety and quality; in addition, pathogenesis of food bacteria is related to a cell to cell communication mechanism called *quorum sensing* (QS). Oregano essential oil (OEO) has proved to be useful as food antimicrobial; however, its food applications can be compromised by the volatile character of its active constituents. Therefore, formulation of edible films containing OEO can be an alternative to improve its food usages. QS inhibitory activity of OEO and pectin-OEO films was evaluated using *Chromobacterium violaceum* as bacterial model. Additionally, antibacterial activity was tested against *Escherichia coli* O157:H7, *Salmonella* Choleraesuis, *Staphylococcus aureus,* and *Listeria monocytogenes*. OEO was effective to inhibit bacterial growth at MIC of 0.24 mg/mL for all tested bacteria and MBC of 0.24, 0.24, 0.48, and 0.24 mg/mL against *E. coli* O157:H7, *S.* Choleraesuis, *S. aureus*, and *L. monocytogenes*, respectively. Pectin-films incorporated with 36.1 and 25.9 mg/mL of OEO showed inhibition diameters of 16.3 and 15.2 mm for *E. coli* O157:H7; 18.1 and 24.2 mm for *S.* Choleraesuis; 20.8 and 20.3 mm for *S. aureus*; 21.3 and 19.3 mm for *L. monocytogenes,* respectively. Pectin-OEO film (15.7 mg/mL) was effective against *E. coli* O157:H7 (9.3 mm), *S. aureus* (9.7 mm), and *L. monocytogenes* (9.2 mm), but not for *S.* Choleraesuis. All concentrations of OEO (0.0156, 0.0312, 0.0625 and 0.125 mg/mL) and pectin-OEO films (15.7, 25.9 and 36.1 mg/mL) showed a significant anti-QS activity expressed as inhibition of violacein production by *C. violaceum*. Additionally, the application of pectin-OEO films was effective reducing total coliforms, yeast, and molds of shrimp and cucumber slices stored at 4°C during 15 d. These results demonstrated the potential of pectin films enriched with OEO as food related microorganisms and QS inhibitors.

## INTRODUCTION

Despite advances in food safety, foodborne illness remains common around the world; in the United States more than 9 million people each year have suffer foodborne diseases caused by major pathogens: *Escherichia coli* O157:H7, *Salmonella*, *Campylobacter,* and *Listeria monocytogenes* (Scallan et al., 2011; CDC, 2014). The resistance and pathogenesis of these bacteria could be related to intercellular communication mechanisms like *quorum sensing* (QS), which is based on the synthesis, exchange and perception of small signaling molecules at given cellular densities (Dong and Zhang, 2005; Alvarez et al., 2012). This mechanisms allow Gram (+) and Gram (-) bacteria to regulate some physiological activities, such as virulence, competition amongst populations, conjugation, antibiotic production, motility, sporulation, and biofilm formation (Miller and Bassler, 2001; Truchado et al., 2012; Nazzaro et al., 2013). It has been hypothesized a regulation of food bacterial proliferation by QS, and its inhibition could be a good strategy to assure food safety and quality(Rasch et al., 2005).

Recently, plant essential oils have shown antimicrobial and anti-QS activity (Jaramillo-Colorado et al., 2011; Kerekes et al., 2013). Oregano essential oil (OEO) has been shown to possess the highest antimicrobial activity compared with other essential oils (Burt, 2004), and has been reported as a QS inhibitory extract (Nagy, 2010). OEO has been effective inhibiting the microbial growth of some food pathogens, such as *Staphylococcus aureus*, *E. coli*, *Bacillus subtilis,* and *Saccharomyces cerevisiae* at 0.625 μL/mL (Lv et al., 2011). Additionally, reduced *Salmonella* Newport inoculated in romaine and iceberg lettuce (0.7–4.8 log CFU/g), spinach (0.7–4.9 log CFU/g), and baby spinach (0.5–4.7 log CFU/g; Moore-Neibel et al., 2013). Carvacrol (one the main compounds of OEO) was effective inhibiting the growth and survival of *L. monocytogenes, Aeromonas hydrophila,* and* Pseudomonas fluorescens* showing MIC values of 0.6, 0.6, and 2.5 μL/mL, respectively. Also the exposition of vegetables to these MICs caused the reduction in bacterial counts (<2 log CFU/g; de Sousa et al., 2012). Additionally, it has been reported that carvacrol possess anti-QS activity against *Chromobacterium violaceum* at < 0.05 mM (Ojo-Fakunle et al., 2013). Woertman (2014) reported that carvacrol at concentrations of 0.2 and 0.4 mM reduced violacein production induced by QS of *C. violaceum,* without affecting bacterial viability. However, the high volatile character of the oil constituents complicates its uses in food matrices. In some cases food matrices do not allow the direct addition of this type of oils and other strategies must be evaluated.

Edible films could be useful to carry and stabilize volatile food additives (Min et al., 2005), and additionally act as barriers to oxygen permeability, moisture loss and lipid migration, maintain firmness and sensory attributes of the coated food (Oms-Oliu et al., 2008; Moalemiyan et al., 2012; Ferrari et al., 2013). Furthermore, pectin films added with cinnamon leaf oil and applied in fresh-cut peach and grapes decreased microbial growth and increased the antioxidant status of the treated fruit (Ayala-Zavala et al., 2013; Melgarejo-Flores et al., 2013). For this reason, the objective of this work was to evaluate the *in vitro* antibacterial and anti-QS activities of pectin films added with OEO, and their antimicrobial activity on coated shrimp and cucumber.

## MATERIALS AND METHODS

### PECTIN-OEO FILMS FORMULATION

Pectin-OEO films were prepared using 3 g of citrus peel pectin (≥74% galacturonic acid and methoxy groups ≥ 6.7%, Sigma, St. Louis, MO, USA), 0.99 mL of glycerol, and OEO (*Lippia graveolens* Kunt, ORE Procesadora de oregano silvestre, Chihuahua, Mexico) at different concentrations (0, 15.7, 25.9, and 36.1 mg/mL), the components were dissolved in 100 mL of distilled water and homogenized for 15 min for edible films and coatings (Ayala-Zavala et al., 2013; Melgarejo-Flores et al., 2013). For films, 15 mL of each forming solution were poured in Petri dishes and dried at 60°C for 24 h: the dried films were separated from the dishes and used for antibacterial assays.

### ANTIBACTERIAL ACTIVITY OF OEO AND PECTIN-OEO FILMS AGAINST PATHOGENIC BACTERIA

The antibacterial activity of the OEO and pectin-OEO films were tested against *E. coli* O157:H7 (ATCC 43890), *S. enterica* subsp.* enterica* serovar Choleraesuis (ATCC 7001), *S. aureus* (ATCC 6538), and *L. monocytogenes* (ATCC 7644). The inoculums were prepared using a 16 h culture adjusted by reference to an OD of 0.1 at 600 nm using a microplate reader (Fluostar Omega, BMG Labtech, Chicago, IL, USA), and further diluted with Mueller Hinton (MH) broth to achieve approximately 1.25 × 10^8^ CFU/mL. Disk diffusion method was carried out to test the antibacterial activity of the films. With the aid of a moist sterile swab the inoculums were spread on plates of MH agar, left to dry for 15 min and incubated at 37°C. Subsequently, pectin-OEO disks (0.5 cm of diameter) were placed on the inoculated plates and incubated at 37°C for 24 h. After incubation, the inhibition zones were measured to determine antibacterial activity, subtracting the film diameter (mm). The resulting diameters of the inhibition zones were compared with control pectin disks without OEO. Broth microdilution method was developed to evaluate the antibacterial activity of OEO. Five μL of inoculums and 295 μL of MH broth enriched with OEO (at concentrations ranging 0.1–1 mg/mL) were taken and placed in sterile 96-well microplates (Costar 96), and incubated during 24 h at 37°C. The lowest concentration of the OEO at which the tested bacteria did not show visible growth was taken as the minimal inhibitory concentration (MIC); additionally, the minimal bactericidal concentration (MBC) was determined as the lowest tested concentration of OEO to eliminate the inoculums; both MIC and MBC were expressed as mg/mL.

### INHIBITION OF *C. violaceum* QS BY THE PRESENCE OF OEO AND PECTIN-OEO FILMS

*C. violaceum* (ATCC 12472) was used to determine the anti-QS effect of the oil and films. This bacterial model uses an intercellular communication response by the production of a purple pigment (violacein), induced by the presence of acyl homoserine lactones; analyzing the production of the pigment it is possible to evaluate the interference of a given substance in this process (Adonizio et al., 2006; Truchado et al., 2009). With this in mind, the inoculum of *C. violaceum* was grown aerobically in Luria-Bertani (LB) broth and incubated at 30°C for 18 h to obtain an OD of 0.1 at 600 nm (1.25 × 10^8^ CFU/mL).

#### Disk diffusion assay

LB agar plates were spread with 0.1 mL of *C. violaceum* inoculum, and then, disks of pectin-OEO films (0, 15.7, 25.9, and 36.1 mg/mL), and the free oil impregnated in paper disks (0, 6.25, 12.5, and 25 mg/mL) were placed on LB plates and incubated at 30°C during 24 h. Growth and/or pigment inhibition around the disk was recorded after incubation following the methodology described by Zahin et al. (2010). *C. violaceum* growth inhibition (clear halo) was measured as radius (r1) in mm while both growth and pigment inhibition (clear plus turbid halo) was measured as radius (r2) in mm. The pigment (QS) inhibition was determined by subtracting bacterial growth inhibition radius (r1) from radius (r2) thus QS inhibition = (r1–r2) to check the inhibition of violacein production around the disks. This experiment was carried out by triplicate.

#### Quantification of pigment production

The violacein production of *C. violaceum* exposed to OEO and pectin-OEO films was quantified. Ten mL of LB broth containing sub-inhibitory concentrations of OEO (0.0156, 0.312, 0.625, and 0.125 mg/mL) and pectin-OEO films (0.25, 0.5, 1, and 2 mg/mL of pectin films added with 15.7, 25.9, and 36.1 mg/mL of OEO) were inoculated and incubated at 30°C for 24 h. The quantification of violacein production was carried out following the protocol described by Choo et al. (2006), where 1 mL of culture from each sample was centrifuged at 13,000 rpm for 10 min to precipitate the insoluble violacein. Then, the culture supernatant was discarded, and the pellet was solubilized in 1 mL of dimethylsulfoxide, vortexed until the violacein was extracted, and centrifuged at 13,000 rpm for 10 min to remove cells. OD of each violacein containing supernatant was measured at 585 nm, results were expressed as percentage of violacein production. The control of each experiment was LB medium without OEO or films; pigment production of control sample was set as 100%. LB plus pectin without OEO was confirmed as negative control. The viability of *C. violaceum* was analyzed taking 1 mL serially diluted of each treatment (OEO and pectin-OEO films), and spread onto LB agar plates at 30°C for 24 h. The bacteria counts were expressed as log CFU/mL, this assay was performed by triplicate.

### ANTIMICROBIAL ACTIVITY OF PECTIN-OEO COATINGS ON SHRIMPS AND CUCUMBERS

Fresh shrimps (*Litopenaeus vannamei*) and cucumbers (*Cucumis sativus* L.) were selected free from signs of decay from local market. Shrimps and cucumber slices (slices of 0.5 cm of cucumber) were coated by immersion during 2 min, in pectin-OEO solutions (0, 15.7, 25.9, and 36.1 mg/mL) prepared as described before, the excess of the coating solutions was drained. The coated food was let to dry at 25°C during 30 min, and then packed in polypropylene trays and stored at 4°C during 15 days. Total coliforms, yeast and molds counts of the coated food were recorded according to the U.S. Food and Drug Administration Bacteriological Analytical Manual methodology (FDA, 2014). The coated foods (10 g) were diluted (1:9) in a solution containing 0.1 g/100 mL of peptone and 0.5 g/100 mL of sodium chloride and homogenized for 1 min. Subsequently, 10-fold dilutions were also made in this diluent. Each dilution was plated in triplicate in violet red bile and potato dextrose agars, for total coliforms, yeast and molds, respectively. Results were expressed as log CFU/g of the sample.

### STATISTICAL ANALYSIS

A completely randomized experimental design was applied to all experiments. The antimicrobial effect of OEO and pectin-OEO films (MICs and diameter of inhibition zones against *E. coli, S.* Choleraesuis, *L monocytogenes*, and *S. aureus*) and the QS inhibition (inhibition radios, pigment production, and viability of *C. violaceum*) were evaluated. In addition, the effect of pectin-OEO coatings on shrimp and cucumber slices was tested. An analysis of variance (ANOVA) was performed (*p* ≤ 0.05) to estimate significant differences between the treatments, and Tukey’s mean test was used for comparison (*p* ≤ 0.05) using the NCSS 2007.

## RESULTS

### ANTIMICROBIAL ACTIVITY OF OEO AND PECTIN-OEO FILMS

**Table 1** shows the antibacterial activity of OEO against pathogenic bacteria. OEO was effective to inhibit bacterial growth at a MIC of 0.24 mg/mL for all tested bacteria; and showed MBC of 0.24, 0.24, 0.48, and 0.24 mg/mL against *E. coli* O157:H7, *S.* Choleraesuis, *S. aureus*, and *L. monocytogenes*, respectively. **Table 2** shows that pectin-film incorporated with 36.1 and 25.9 mg/mL of OEO were more effective, showing inhibition zones with diameters of 16.3 and 15.2 mm for *E. coli* O157:H7; 18.1 and 24.2 mm for *S.* Choleraesuis; 20.8 and 20.3 mm for *S. aureus*; 21.3 and 19.3 mm for *L. monocytogenes,* respectively. The pectin-OEO film (15.7 mg/mL) was effective against *E. coli* O157:H7 (9.3 mm), *S. aureus* (9.7 mm), and *L. monocytogenes* (9.2 mm), but no inhibition zones were observed for *S.* Choleraesuis.

**Table 1:** Minimum inhibitory concentration (MIC; mg/mL) and minimum bactericidal concentration (MBC; mg/mL) of OEO against pathogenic bacteria.

	*Escherichia coli*	*Salmonella* Choleraesuis	*Staphylococcus aureus*	*Listeria monocytogenes*
MIC	0.24	0.24	0.24	0.24
MBC	0.24	0.24	0.48	0.24

*Values are mean of three replicates*.

**Table 2:** Inhibition zones of pectin-OEO films against pathogenic bacteria.

OEO in pectin films (mg/mL)	Inhibition zone diameter (mm)			


	*E. coli*	*S.* Choleraesuis	*S. aureus*	*L. monocytogenes*
0	0	0	0	0
15.7	9.3 ± 0.11	0	9.7 ± 0.057	9.2 ± 0.04
25.9	16.3 ± 0.27	18.1 ± 0.29	20.8 ± 0.08	21.3 ± 0.24
36.1	15.2 ± 0.052	24.2 ± 0.18	20.3 ± 0.06	19.3 ± 0.104

*Values are mean ± SD of three replicates*.

### INHIBITION OF *C. Violaceum* CELL TO CELL COMMUNICATION EXPOSED TO OEO AND PECTIN-OEO FILMS

**Table 3** shows the effectiveness of different concentrations of OEO and pectin-OEO films to inhibit QS of *C. violaceum*. According to inhibition radios, both OEO and pectin-OEO films exhibited concentration dependent anti-QS activity. OEO tested at 6.25 and 12.5 mg/mL exerted a significant pigment inhibition without affecting *C. violaceum* growth. At elevated concentration as 25 mg/mL, this extract demonstrated antibacterial activity along with the anti-QS activity (**Table 3**). With regards to film plus OEO, exerted a higher pigment inhibition when compared with free OEO. The highest concentration of OEO enriched film tested, 36.1 mg/mL, inhibited bacterial growth along with pigment production. These differences may be due to the presence of higher concentrations of OEO in the films.

**Table 3:** Anti-QS activity of OEO and pectin-OEO films by disk diffusion method using *C. violaceum* as indicator strain.

		Inhibition zones against *C. violaceum* (radios in mm)		
	Concentration (mg/mL)	Growth inhibition (r1)	Growth + pigment inhibition (r2)	QS inhibition (r2–r1)



OEO	0	ND	ND	ND
	6.25	ND	4.6 ± 0.29	4.6 ± 0.29
	12.5	ND	5.6 ± 0.29	5.6 ± 0.29
	25	5.0 ± 0.00	10.8 ± 1.04	5.8 ± 1.04
Pectin-OEO film	0	ND	ND	ND
	15.7	ND	25.6 ± 0.76	25.6 ± 0.76
	25.9	ND	30.5 ± 2.78	30.5 ± 2.78
	36.1	4.8 ± 0.29	26.8 ± 1.60	22.0 ± 1.80

*Values of radios are mean ± SD of three replicates. ND, not detected inhibition*.

**FIGURE 1:**
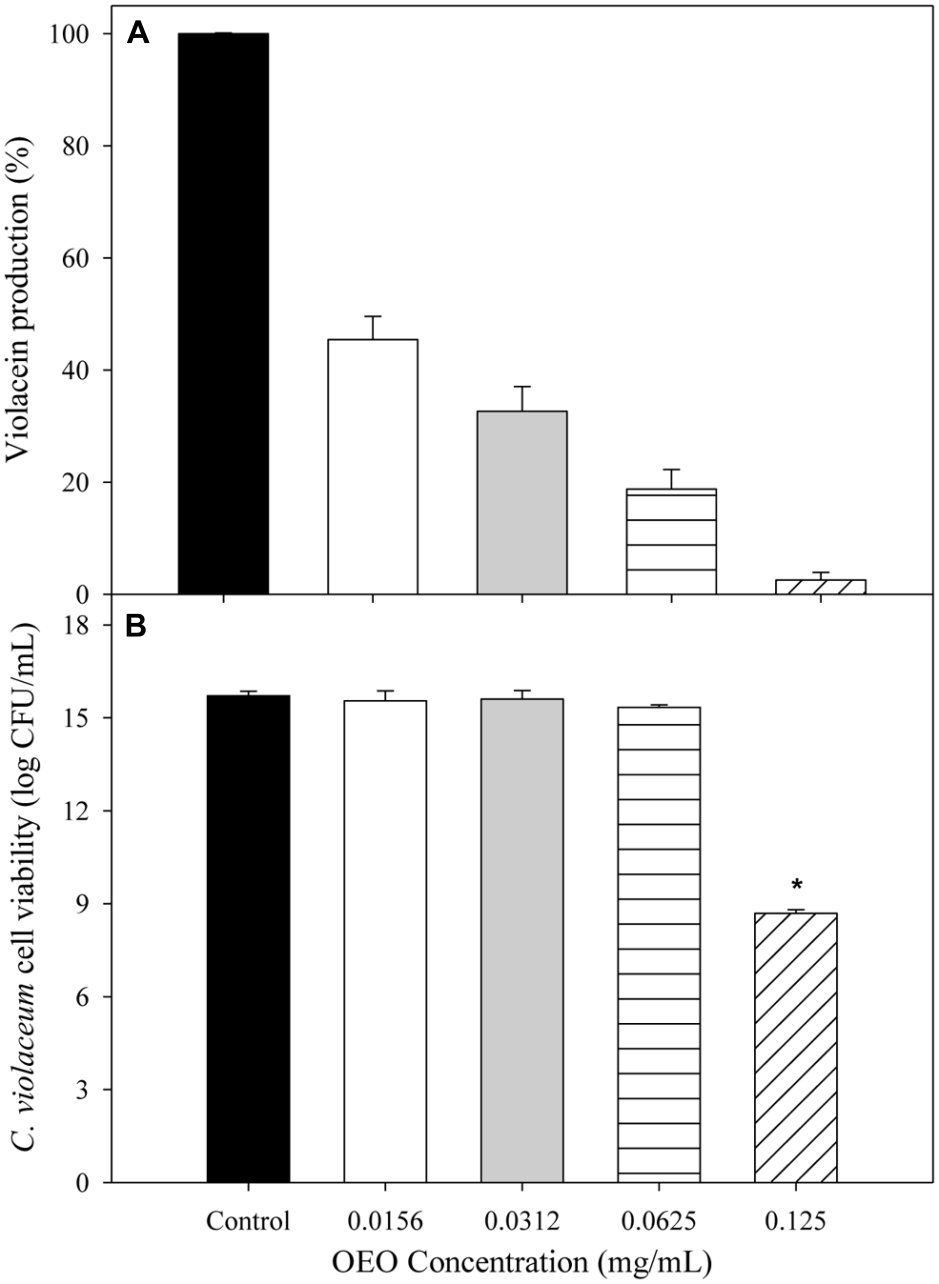
**(A)** Inhibition of violacein production of *C. violaceum* exposed to OEO at different concentrations. **(B)** *C. violaceum* viability after incubation in LB broth enriched with different concentrations of OEO. *Significantly different when compared to control (*p* < 0.05).

**FIGURE 2:**
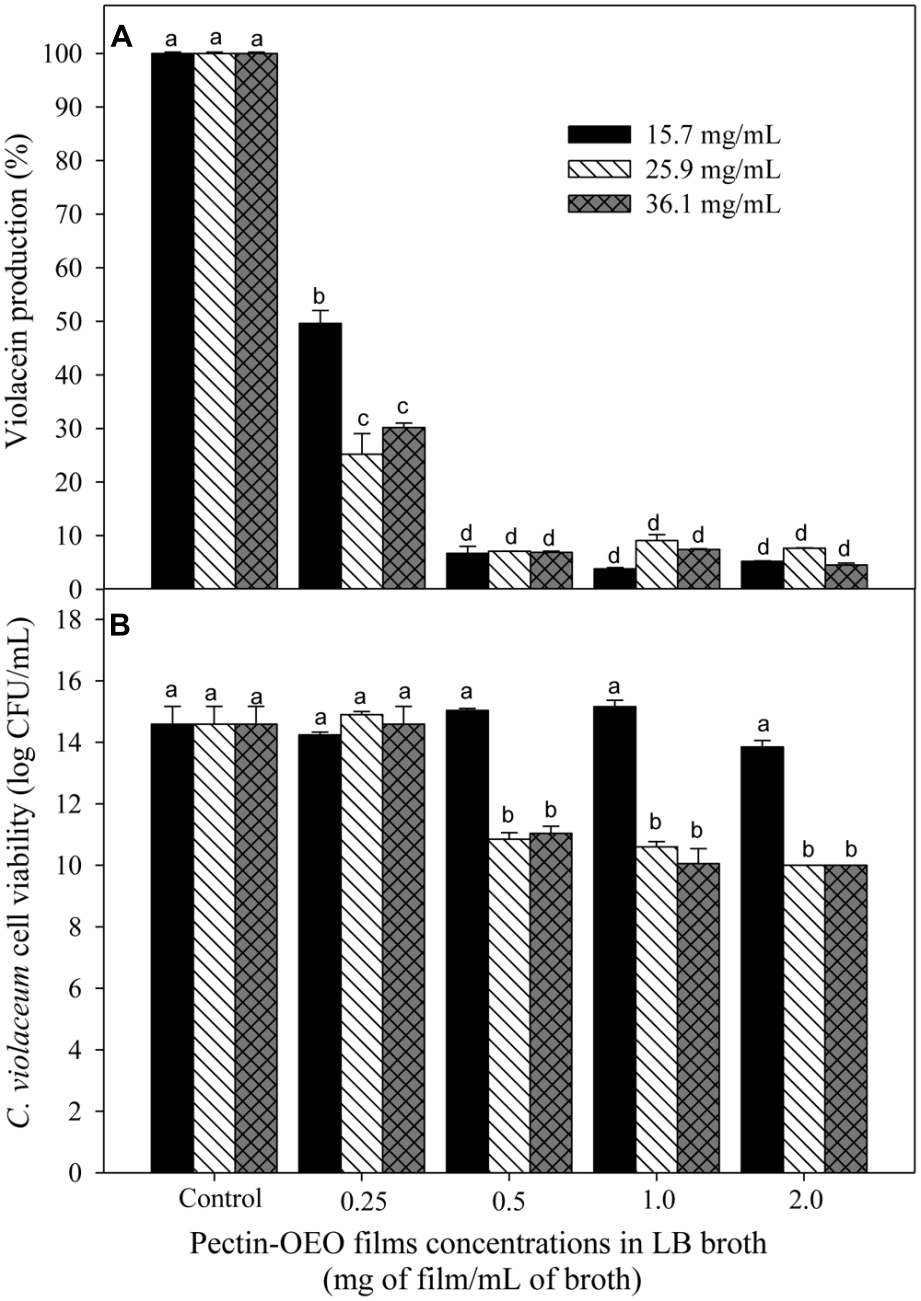
**(A)** Inhibition of violacein production of *C. violaceum* exposed to different concentrations of OEO enriched pectin films in LB broth, using films prepared by incorporation of 15.7, 25.9, and 36.1 mg/mL of OEO. Different letters among bars indicated significant differences (*p* < 0.05). **(B)** *C. violaceum* cell viability after incubation in LB broth enriched with different concentrations of OEO enriched films in LB broth. Different letters among bars indicated significant differences (*p* < 0.05).

**Figure 1A** shows the capacity of OEO to inhibit violacein production from *C. violaceum* exposed to different concentrations (0.0156, 0.0312, 0.0625, and 0.125 mg/mL). To evaluate whether the inhibition of violacein production owed to the QS mechanism inhibition or to microbial growth reduction, the *C. violaceum* concentration was also determined. An inverse relationship between pigment production and the applied OEO concentrations was noticed, compared with control samples. All the tested OEO concentrations showed a significant drop in violacein production even the lowest one (0.0156 mg/mL) which reduced pigment production by more than 50%. In addition, the cell viability (**Figure 1B**) of *C. violaceum* was not affected at concentrations of 0.156, 0.032, and 0.0625; only the highest concentration (0.125 mg/mL) inhibited the growth reducing the bacterial counts (7 log CFU/mL) after the incubation period. Similarly, pectin-OEO films showed a significant inhibition of violacein production (**Figure 2**) compared with controls. All pectin films added with 15.7, 25.9, and 36.1 mg/mL of OEO were tested at concentrations of 0.25, 0.5, 1, and 2 mg/mL, and all of them were effective reducing the violacein production. No differences were observed among the concentrations of 0.5, 1, and 2 mg/mL of pectin films added with 15.7, 25.9, and 36.1 mg/mL of OEO showing the highest inhibition percent (more than 90%) of the violacein production. With respect to the effect of the pectin-OEO films on the *C. violaceum* viability (**Figure 2B**), it was observed that for all the pectin-OEO films the concentration of 0.25 mg/mL inhibited QS without affecting the cellular viability, in addition, the concentrations of 0.5, 1, and 2 mg/mL of the pectin films added with 15.7 mg/mL of OEO did not affect cellular viability and affected violacein production. On the other hand, the inhibition on pigment production exerted by 25.9 and 36.1 mg/mL pectin-OEO films applied at ≥ 0.5 mg/mL showed significant (*p* < 0.05) antibacterial effect instead of a blockage of QS mechanism.

### EFFECT OF PECTIN-OEO COATINGS ON THE MICROBIAL GROWTH OF SHRIMP AND CUCUMBER

**FIGURE 3:**
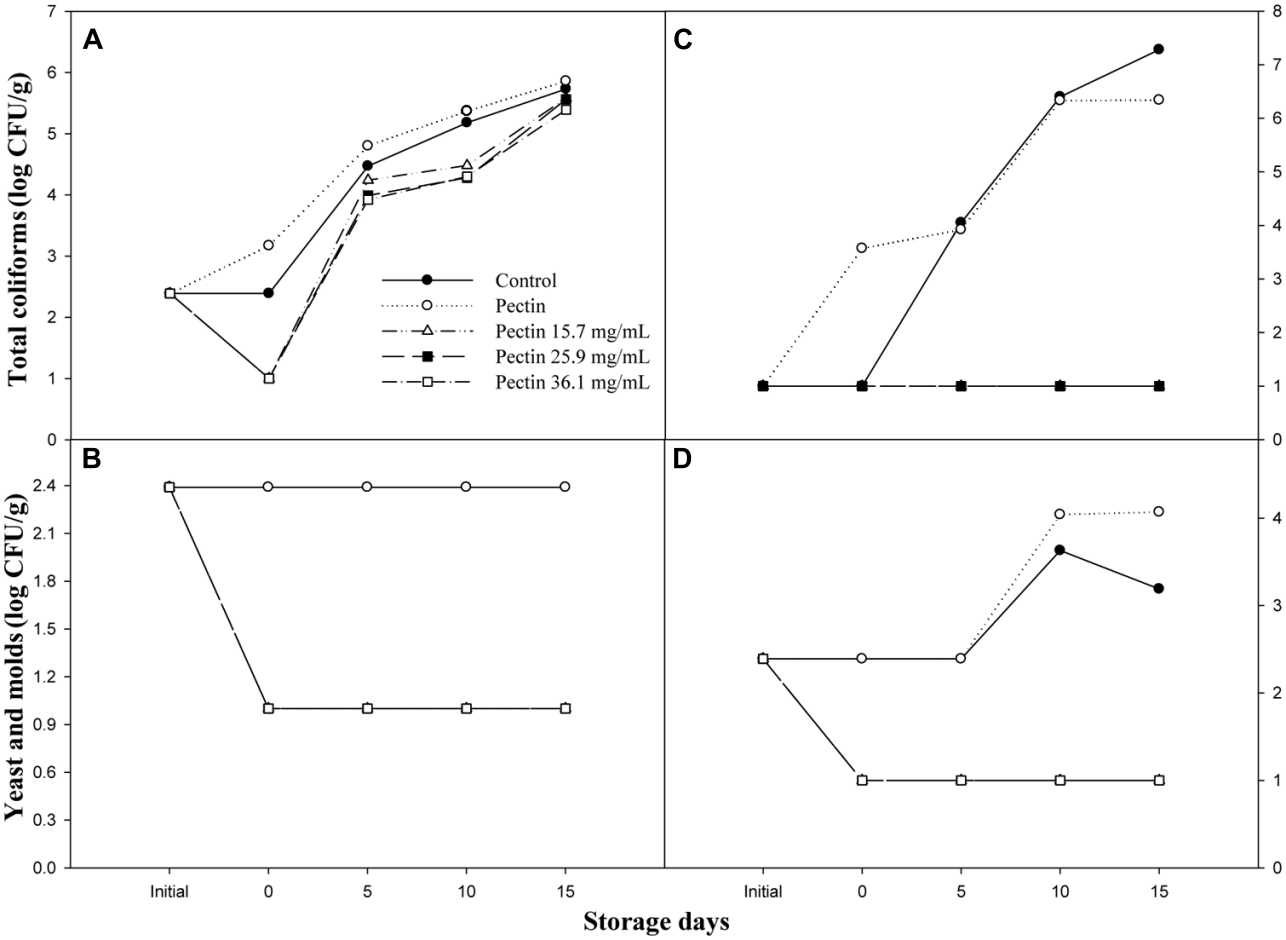
**Effect of OEO on the microbial load (log CFU/g), **(A)** total coliforms and **(B)** yeast and molds in shrimp; **(C)** total coliforms and **(D)** yeast and molds in sliced cucumber throughout 15 days of storage at 4°C**.

**Figure 3** shows the effectiveness of pectin-OEO coatings to reduce the growth of total coliforms, yeast and molds of shrimp and sliced cucumber stored at 4°C during 15 days. Pectin-OEO coatings of 15.7, 25.9, and 36.1 mg/mL were effective reducing 1.39 log CFU/g of total coliforms with respect to control fruit at day 0. All treatments showed an increment in total coliforms load during the storage time; however, the pectin-OEO coated shrimp showed lower load during the storage (**Figure 3A**). Similarly, the pectin-OEO coated shrimp (**Figure 3B**) caused a general reduction of total yeast and molds of 1.4 log CFU/g with respect to control and pectin films (2.39 log CFU/g) and this difference remained throughout the storage time. The effect of pectin-OEO coatings on the microbial load of fresh-cut cucumber (Figures 3C,D) was similar to that observed for shrimps. The three concentrations of pectin-OEO coatings maintained the total coliforms at 1 log CFU/g during the storage time (C) compared with the control and pectin coating that showed 7.28 and 6.34 log CFU/g at the end of the storage. Similarly to the effect observed in shrimp, total yeast and molds load of sliced cucumber coated with the pectin-OEO formulations (**Figure 3D**) maintained the load at 1 log CFU/g compared with control and pectin coated fruit that shown 3.19 and 4.07 log CFU/g throughout the storage. These results demonstrated that the incorporation of OEO in pectin coatings reduced the microbial growth of the studied food, showing a protective effect over the storage time.

## DISCUSSION

Oregano essential oil antimicrobial activity has been widely reported in the literature against some foodborne pathogens. In our study, the MIC for all tested bacteria was different to those reported in previous studies (Burt and Reinders, 2003). OEO from *Origanum vulgare* presented a bactericidal effect against *E. coli* O157:H7 when applied at 0.57 mg/mL (Burt and Reinders, 2003). On the other hand, Lambert et al. (2001) reported the OEO MIC as 0.575 mg/mL against *S. aureus*. The differences found with previous works could be attributed to several factors like development stage, variety, ecological conditions, and other plant factors (Özcan and Erkmen, 2001). With respect to inhibition of pectin films added with OEO, other studies shown higher inhibition zones as reported by Du et al. (2009) which evaluated the antimicrobial activity of OEO in pectin films against *E. coli* O157:H7, *S. enterica* and *L. monocytogenes*. They found that pectin film incorporated with OEO presented a high antimicrobial activity. In addition, main components of OEO such as carvacrol added to pectin films presented antimicrobial activity against *E. coli* O157:H7 (Du et al., 2008). On the other hand, Seydim and Sarikus (2006) tested whey protein films added with OEO at 2% and showed inhibition zones of 49.1, 65.4, 62.0, 61.4, and 34.4 mm against *E. coli* O157:H7, *S. aureus*, *S. enteritidis*, *L. monocytogenes,* and *Lactobacillus plantarum*, respectively. These higher values can be an indicative of the effect of the polymer matrices used in the film formulation. The antibacterial efficacy of the OEO in different food matrices reflects its potential uses in the food industry against pathogenic bacteria.

Some studies have demonstrated the potential use of different plant extracts with anti-QS activity (Alvarez et al., 2012). Loss of purple pigment in *C. violaceum* is an indicative of QS inhibition by the bioactive products. The results obtained in this study reflects the potential of OEO free or incorporated within pectin films at low concentrations as promising QS inhibitors, considering that were able to interrupt intercellular communication inhibiting the violacein production without affecting growth inhibition (Choo et al., 2006). The observed anti-QS effect of the OEO could be attributed to its bioactive constituents such as carvacrol. Vattem et al. (2007) reported that carvacrol reduced violacein production of *C. violaceum* up to 8 mM without affecting viability. These authors associated the pigment inhibition of carvacrol to the reduction in gene expression coding for the synthesis of acyl homoserine lactones, as described before, it is a key signaling molecule in the QS. Different mechanisms have been proposed to explain the interference of QS depending processes induced by natural products: (i) inhibition of signal molecule biosynthesis or acyl homoserine lactone signal reception (Rasmussen et al., 2005; Vattem et al., 2007), and the ii) enzymatic inactivation and biodegradation of QS molecules (Defoirdt et al., 2004). Gao et al. (2003), reported that the interruption of bacterial QS by plant extracts has been barely studied, and highlighted its importance as controller agents for microbial pathogenesis. These findings reflects the potential of free OEO or incorporated within pectin films at low concentrations as promising QS inhibitors, considering that were able to interrupt intercellular communication inhibiting the violacein production without causing growth inhibition (Choo et al., 2006).

Previous studies have been reported the effectiveness of the application of coatings enriched with essential oils in foods (Ravishankar et al., 2009). Pectin films enriched with carvacrol at 3% showed a high reduction against *S. enterica* and *E. coli* O157:H7 (4.3–6.8 log CFU/g) inoculated in chicken breasts (Ravishankar et al., 2009). In another study, 1% of OEO incorporated into a calcium caseinate WPI-carboxymethyl cellulose film was found to be effective against *E. coli* O157:H7 and *Pseudomonas* spp. on the surface of beef muscle pieces (Oussalah et al., 2004). On the other hand, pectin coatings added with other essential oils to prevent deterioration of different vegetable tissues have been widely used; e.g., Melgarejo-Flores et al. (2013) reported a significant inhibition of microbial growth of grapes after application of pectin coatings with cinnamon leaf oil at concentration of 36.1 mg/mL, compared controls and coated grapes only with pectin, which showed the highest spoilage after 15 days at 10°C. Similarly, Ayala-Zavala et al. (2013), applied the same coatings on fresh-cut peaches and observed growth inhibition of *E. coli* O157:H7, *S. aureus,* and *L. monocytogenes* at a concentration of 36.1 mg of cinnamon leaf oil per mL of pectin solution (30 mg/mL).

## CONCLUSION

Oregano essential oil incorporated within edible films or coatings showed an inhibitory effect on the QS of *C. violaceum*, inhibiting the intercellular communication process. In addition, the pectin-OEO mixtures showed antibacterial effect against food pathogenic and spoilage microorganisms. These results can be useful to study the effect of the treatments on the pathogenesis of the studied microorganisms. Therefore, it is highlighted the potential of the studied pectin-OEO edible films and coatings to be used as antimicrobial agent to assure food safety and quality.

## Conflict of Interest Statement

The authors declare that the research was conducted in the absence of any commercial or financial relationships that could be construed as a potential conflict of interest.
